# Application of Ultrasound in Food Science and Technology: A Perspective

**DOI:** 10.3390/foods7100164

**Published:** 2018-10-04

**Authors:** Monica Gallo, Lydia Ferrara, Daniele Naviglio

**Affiliations:** 1 Department of Molecular Medicine and Medical Biotechnology, University of Naples Federico II, Via Pansini, 5, 80131 Naples, Italy; 2 Department of Pharmacy, University of Naples Federico II, Via Domenico Montesano 49, 80131 Naples, Italy; lyferrar@unina.it; 3 Department of Chemical Sciences, University of Naples Federico II, Via Cintia 21, 80126 Naples, Italy; naviglio@unina.it

**Keywords:** ultrasound applications, low power, high power, ultrasound in food technology, ultrasonic extraction, antimicrobial activity of ultrasound

## Abstract

Ultrasound is composed of mechanical sound waves that originate from molecular movements that oscillate in a propagation medium. The waves have a very high frequency, equal to approximately 20 kHz, are divided into two categories (i.e., low-intensity and high-intensity waves) and cannot be perceived by the human ear. Nature has created the first ultrasound applications. Bats use ultrasound to navigate in the dark, and many cetaceans use echolocation to detect prey or obstacles using ultrasound produced by their vocal system. Ultrasound is commonly associated with the biomedical field. Today, ultrasound-based methods and equipment are available to detect organs, motion, tumour masses, and pre/post-natal handicaps, and for kidney stone removal, physiotherapy, and aesthetic cures. However, ultrasound has found multiple applications in many other fields as well. In particular, ultrasound has recently been used in the food industry to develop various effective and reliable food processing applications. Therefore, this review summarizes the major applications of ultrasound in the food industry. The most common applications in the food industry include cell destruction and extraction of intracellular material. Depending on its intensity, ultrasound is used for the activation or deactivation of enzymes, mixing and homogenization, emulsification, dispersion, preservation, stabilization, dissolution and crystallization, hydrogenation, tenderization of meat, ripening, ageing and oxidation, and as an adjuvant for solid-liquid extraction for maceration to accelerate and to improve the extraction of active ingredients from different matrices, as well as the degassing and atomization of food preparations.

## Highlights

Ultrasound (US) is divided into different frequency ranges.

US has been applied in very different fields.

Competitive energy costs and low maintenance make US processes economically profitable.

US techniques have been used in the food industry to analyse and modify foods.

## 1. Introduction

Sound is an oscillation of particles (i.e., atoms and molecules) in an elastic medium. The oscillations displace the particles around the resting position and along the direction of propagation of the wave caused by vibratory movements. These vibrations are produced by a given object, which is called the source of sound, which transmits its movement to the adjacent particles by virtue of the mechanical properties of the medium. When oscillation begins, the particles transmit their movements to neighbouring particles, and these particles in turn transmit their movements to others, causing a local variation in pressure; therefore, a simple vibratory movement propagates mechanically to produce a sound wave (or acoustic wave) [1]. Ultrasound (US) is defined as waves of a mechanical nature that require an elastic medium to propagate [2,3]. Sounds and ultrasounds differ in frequency: sound waves propagate at frequencies audible to the human ear (from 16 Hz to 16–20 kHz), while US waves propagate at frequencies greater than 20 kHz (upper limit of audibility for the human ear) up to frequencies of 10 MHz, which then proceeds to the so-called hypersonic region. Ultrasound, which is composed of mechanical waves, propagate in a medium through the transfer of energy and not of particles; the latter, in fact, simply oscillate around their equilibrium position, with the transfer of energy from one particle to another. The oscillation propagates in the medium in various directions, and therefore, distinguish as longitudinal waves and transverse waves. In longitudinal waves, the oscillatory movement of the particles in the transmission medium is parallel to the propagation direction, while in the transverse waves the movement is perpendicular. The longitudinal (or compression) waves propagate in any medium, while the transverse waves only propagate in solid media. The US wave is a longitudinal wave characterized by the alternation of compression and rarefaction cycles of the medium in which it propagates, which entails variations in the pressure of the medium; energy is transferred from the motion of particles. This process of compression and rarefaction of the particles in the medium and their subsequent collapse is known as the phenomenon of cavitation, the most important effect of high-energy US waves [4,5]. In particular, cavitation is a physical phenomenon that leads to the formation and activity of bubbles (or cavities) inside a liquid when it is subjected to the action of high-speed pressure and depression waves generated by ultrasound waves in an intense ultrasonic field. During the depression phase, numerous bubbles are created inside the liquid. During the second phase of ultrasound compression, the enormous pressure exerted on the bubble decompresses until it implodes, i.e., collapse on itself. The bubbles have a diameter of a few micrometres, while the lifetime of the bubbles is on the order of microseconds. Two types of cavitation have been distinguished: in the first type, known as stable cavitation, the bubble remains stable around an equilibrium size for many compression-decompression cycles; in the second type, known as transitory or unstable cavitation, the bubble grows in a single cycle, doubles its size, and then collapses.

The chemical effects of US vary, and three distinct phases have been identified in the reaction environment: the gaseous environment inside the bubble cavity, the liquid-bubble interface, and the liquid. In the gas phase, pyrolysis reactions occur, such as the pyrolysis of water. In the bubble-liquid zone and the liquid mass, various radicals can form, and the most frequently encountered radical in the aqueous environment is the hydroxide radical OH·, which is highly reactive and readily attacks organic substrates present in the reaction environment or recombines with another OH· radical to form H_2_O_2_. In the interface area, where the temperatures are very high, solute reactions with OH· radicals or thermal degradation reactions may occur. The diffusion of these radicals is mainly due to the disruption of the cavitation bubble and subsequent formation of smaller bubbles. Non-volatile solutes react in the interface zone or in the liquid, and volatile solids enter the bubble and degrade during bubble collapse. The effects of radicals are important because they induce molecular sonolysis and degradation of the solvent and solute structure. In contrast, mechanical effects alter electrochemical processes, modify the properties of certain solids and alter the liquid-liquid and gas-liquid systems by facilitating the formation of solid-state emulsions and solute dispersion in the solvent [6,7]. Historically, US has developed similar to a branch of acoustics. In parallel to the progress in understanding the propagation of acoustic waves, we have witnessed the development of technologies capable of generating US. As described above, US waves are sound waves with a frequency range ranging from 20 kHz to 10 MHz. Further subdivisions within this range have been identified [8]; these subdivisions may in fact have substantially different characteristics, depending on the frequency at which they are generated and the amount of energy generated by the acoustic field. In particular, US power influences chemical reactivity and is grouped into two subfamilies: A) high-energy US characterized by low frequencies (20 kHz–100 kHz), which are used in some food technologies, and intermediate power US processes characterized by medium frequencies (100 kHz–1 MHz); and B) low-energy diagnostic US that is used in physical measurements, mainly for medical and diagnostic uses, and is characterized by high frequencies (5 MHz–10 MHz). Although cavitation is considered an event to be avoided in many fields, other fields exploit cavitation produced in a controlled manner, such as the military, which uses torpedoes that travel at very high speeds, or ultrasonic cleaning systems used to clean small objects, where the implosions of the bubbles clean even the most delicate and unreachable surfaces. In the medical field, controlled cavitation is used to remove kidney stones (lithotripsy), which are precisely crushed through the formation of microbubbles that implode the solid formations inside the kidneys, as well as for diagnosis and antalgic thermal effects [9]. Cavitation is also used in aesthetic medicine to eliminate or reduce adiposity, a technique known as non-surgical non-bloody liposuction (Ultrasound Lipoclasia). In addition, acoustic cavitation has been used to facilitate the delivery of small molecules and macromolecules, including proteins and DNA. Controlled generation of cavitation has also been used for the targeted delivery of drugs to diseased tissues, including the skin, brain, eyes and endothelium [10]. Moreover, US has been employed as a treatment for several diseases, including thromboembolism, arteriosclerosis, and cancer [11,12,13,14]. On the other hand, the study of the propagation of US waves in humans has allowed the construction of stable medical diagnostic instruments used in gynaecology, gastroenterology, angiology, and cardiology, which exploit the return echo resulting from an US wave that propagates inside the human body and is slowed in a different way by the different anatomical structures it crosses [15]. Moreover, precisely because of the different acoustic impedance of the various tissues, US has been used to determine various biological effects, among which the thermal effect was the original use of US in orthopaedics, physiatrics, and sports medicine to inhibit pain and particularly in the aesthetic field to treat localized adiposity and cellulite [16].

**Figure 1:**
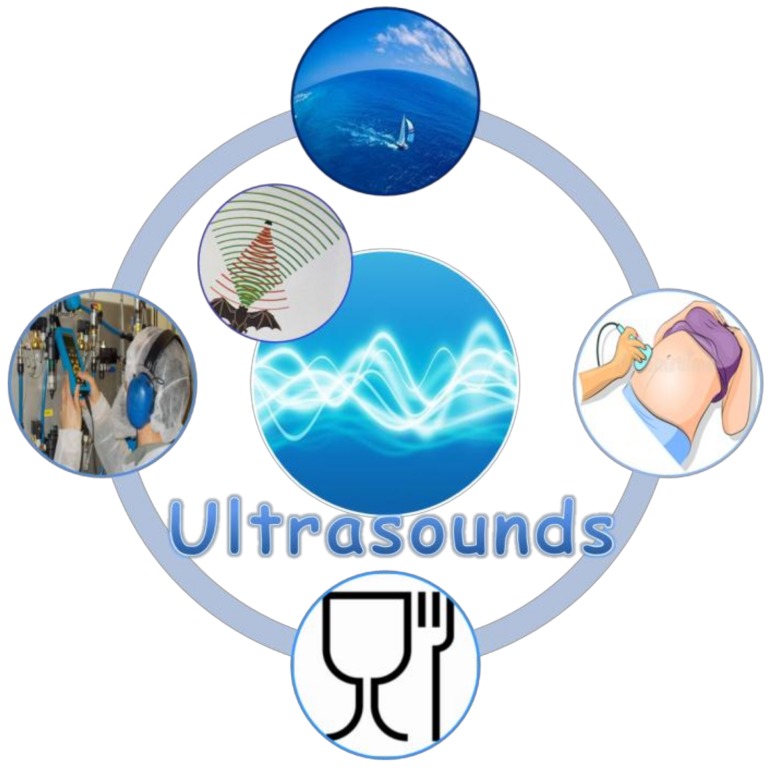
The different fields in which ultrasound (US) has been applied.

In the food sector, the use of US has many advantages. In fact, US has been used to obtain phytocomplex extracts without altering their organoleptic properties, enabling researchers to understand their functional principles. Any solvent can be used for extraction based on the type of extract desired, and the process operates exclusively at room temperature, resulting in remarkable reductions in the required extraction time, instrumentation, energy and human resources. In addition, US use guarantees a reduction in the bacterial content of the final product due to the antibacterial effect of US. Finally, the US extraction process has received biological certification for use in both the food and cosmetic industries [17] (Figure 1).

The fields in which US has been applied exploit both its mechanical and chemical effects. The importance of the two effects varies according to the frequency used [8]. At low frequencies (20–100 kHz), the mechanical effect caused by unstable cavitation predominates, and as the frequency approaches 20 kHz, the bubbles collapse with increasing violence [18]. At medium frequencies (200–500 kHz), the chemical effect is prevalent, as a greater number of bubbles form that collapse less violently. At high frequencies (>1 MHz), both the chemical and physical effects related to cavitation are minimal, while the effect of the acoustic flow is predominant. The high frequencies are typically used to clean delicate objects, which might be damaged in the presence of cavitation.

Table 1 shows some examples of the applications of US to improve and facilitate different types of processes.

However, given the vastness and heterogeneity of the fields in which US is applied, this review exclusively focuses on applications in the food technology sector.

## 2. US in Food Science and Technology

**Figure 2:**
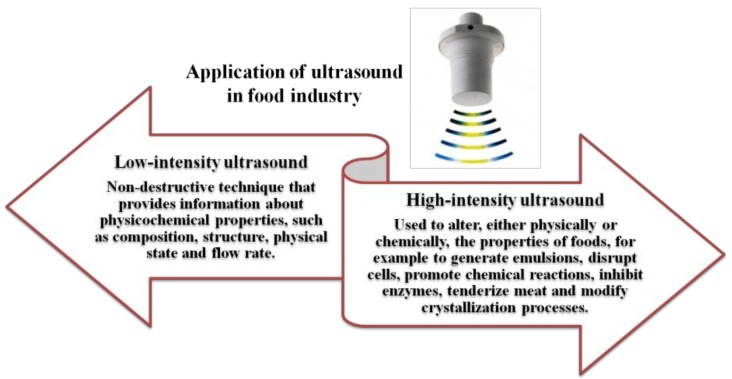
Applications of ultrasound in food analysis and processing.

Over the past few years, the properties of US have aroused increasing interest in the food industry, as the induction of physical and chemical reactions can lead to a strategic advantage in the various stages of processing. Currently, US is considered an emerging and promising technology in the food processing industry, since it produces permanent mechanical, chemical and biochemical changes in liquids (due to intense cavitation) and gases (for the generation of high intensity acoustic fields). Since the 1990s, the use of US has become a technological alternative that is applicable on a large scale in the processing industry [35]. Ultrasound has been applied to food technologies due to its mechanical and/or chemical effects on the processes of homogenization, mixing, extraction, filtration, crystallization, dehydration, fermentation, and degassing through its antifoaming actions, reduction of particle sizes, temporary or permanent modifications of viscosity, modulation of the growth of living cells, cell destruction and dispersion of aggregates, inactivation of microorganisms and enzymes, and sterilization of equipment [24]. Precisely for these reasons, the effects of this technology are considered interesting in the food industry, mainly due to new trends involving consumers shifting towards functional foods. Because US offers several advantages, this form of energy has been applied to improve the qualitative characteristics of high-quality foods and to ensure the safety of a vast variety of foodstuffs, all while minimizing any negative effects on the sensory characteristics of foods. Furthermore, the non-destructive nature of this technology offers several opportunities for the compositional analysis of foods [36]. The interaction of the acoustic energy with a food mainly occurs through a liquid medium since the cavitation and the physical and chemical actions induced by US play an important role in the changes in the quality of a food during its transformation. Indeed, the implosion of bubbles creates an unusual substrate for chemical reactions by mechanically breaking the cell envelope and improving the transfer of intracellular material [8]. At the solid and liquid interface, even the water jet formed by transient cavitation might contribute to some changes in the global properties of a food product. All the chemical and physical effects of US are microscopic; however, the interactions of these chemical and physical reactions induced by cavitation with the food manifests itself through macroscopic changes that are perceived by the consumer in terms of consistency, colour and flavour [8]. High-intensity US has become an effective tool for large-scale commercial applications for approximately a decade, but only recently has the equipment design been optimized and the performance of continuous-flow systems improved. As with all other more innovative processing technologies, high-power US is not a standard technology and therefore must be studied and developed for each type of application [37]. Based on the frequency used and the amplitude of the applied sound wave, a number of physical, chemical and biochemical effects can be used for a variety of applications [38]. High-frequency/low-energy US (diagnostic US) is mainly used as an analytical technique for quality control and processing steps, and for non-destructive inspection that has been applied to determine the concentration, viscosity, and composition of food, among other parameters [22,39,40]. The application of high energy/intensity US (US power) improves the quality of processed foods and results in characteristics similar to the fresh product (colour, consistency, flavour, and nutrients). Few scientific papers have documented a possible correlation between the effects of cavitation and the processing conditions used to predict possible changes in quality. Therefore, only limited conclusions have been drawn about specific foods that have been studied based on the applied parameters [8] (Figure 2).

## 3. US Technology in Different Processes

In the food industry, US is used to control production processes, assess food characteristics, detect defects, control fruit ripening, improve the yield and speed of extraction of food components, and evaluate the conservation state. Ultrasound has been used to improve conventional food processing unit operations by reducing energy and chemical requirements, thus offering a greener option. In food processing, the applications of US are divided into two categories, namely, replacing traditional technologies and assisting traditional technologies. In the latter case, the use of US to enhance various food processes, such as extraction, freezing, thawing, brining, oxidation, filtration, and drying/dehydration, increases the processing efficiency and improves the disadvantages of traditional technologies used during processing [23].

An overview of US applications in different processes is presented below.

Extraction: In the literature, numerous papers have reported the application of US to reduce the extraction time of natural compounds that would normally take hours or days with conventional techniques. Different classes of compounds, such as aromas, polyphenols, organic substances and minerals, have efficiently been extracted from a variety of matrices using US. The mechanical effects facilitate greater penetration of the solvent into the cells, improving the transfer and the cavitation effect, which causes the cell walls to break and release their contents into the medium [41,42]. Therefore, with this technology, higher yields are obtained in less time with lower processing temperatures [43]. Moreover, several applications have shown that US-assisted extraction (UAE) represents an ecological and economically viable alternative to conventional techniques for food and natural products. The main benefits are decreases in extraction and processing times, the amount of energy and solvents used, unit operations, and CO_2_ emissions [22]. Therefore, the technique has also been used to modify foods and create novel food products, which is not possible using conventional food processing technologies [44]. Moreover, due to its high sensitivity and versatility, US methodology has facilitated the identification and quantification of organophosphorus and triazine pesticides in wine samples. In particular, pesticides have been extracted using ultrasound-assisted dispersive liquid-liquid microextraction (USVADLLME) [45]. A recent review focuses on the recently developed applications of phthalate ester (PAE) extraction procedures and subsequent Gas Chromatography-Mass Spectrometry (GC-MS) analysis to food matrices. Several papers have been published in this area, highlighting different sample preparation/extraction methods [46]. Another application of US enabled the determination of phthalates, which are possible endocrine disruptors, in baby food products using a method based on ultrasound-vortex-assisted liquid-liquid microextraction coupled with gas chromatography-ion trap mass spectrometry (GC/IT-MS) [47].

Emulsion: Emulsions and dispersions often contain surfactants to increase stability. The surfactants inhibit the agglomeration of the dispersed material in the liquid phase. In fact, surfactants form a layer around each particle. However, the same surfactants can encapsulate gas bubbles suspended in the liquid phase, which are substantially stabilized. In this way, the surfactant is consumed, the quality of the emulsion or dispersion is reduced, and erroneous readings of the particle size are generated. Liquids are simply degassed by sonication to reduce the problem of stabilized gas bubbles, reducing the number of bubbles and favouring the emulsion. The implosion of a cavitation bubble along the contact surface between two immiscible liquids generates a highly stable emulsion, even at low energy [48,49]. This effect is frequently exploited in the petrochemical, chemical, cosmetic, and pharmaceutical sectors. In the food sector, low-frequency and high-energy US waves are also specifically used to generate emulsions, mayonnaise, fruit juices, ketchup, and homogenized milk [50].

Crystallization: High-intensity ultrasound influences the crystallization process through the initiation of nucleation by controlling the development and the formation of small crystals [19,51]. US-assisted freezing reduces the time required to form crystals and guarantees a greater homogeneity, reducing cell damage, and preserving product integrity [52]. By applying US in batch mode, i.e., sonication, the sugar crystallization process is controlled by facilitating the formation of numerous nuclei from a sugar solution, and the nuclei are then enhanced by the formation of well-defined crystals. Thus, crystallization-resistant nucleation has been initiated in sugars such as D-fructose and sorbitol, which are important sugars in the formulation of candy, confetti, cream, and chocolate [19]. This physical crystallization process occurs without the introduction of foreign substances. In ice cream production, US is used on mixtures of milk, powdered milk, cream, vegetable fats, sugars, emulsifiers, various additives, fruits, flavours, and dyes that must be homogenized, pasteurised and slowly agitated during the freezing process to prevent the formation of large ice crystals. Sonication is applied during freezing to improve the quality of ice cream. Ice crystals with a uniform size and distribution are important contributors to product quality, and nucleation is the most important factor controlling the formation and distribution of crystals during freezing. In this stage, air is also injected in quantities proportional to water and the solids present in the mixture to achieve a creamy consistency, and high-power US improves the quality of the ice cream while maintaining the size of the crystals and preventing the formation of a frozen crust on the surface. Moreover, shorter freezing times enhance the efficiency and capacity of the production process, in addition to producing a better ice cream consistency and creamy taste [20].

Filtration: The vibrational energy generated by US allows the particles to be maintained in suspension and continue to move, leaving the surfaces of the filters free and facilitating the passage of the solvent into the pores. It also creates a frictionless surface that allows the liquid or smaller particles to pass through more easily, thus improving flow and reducing process times. A further advantage is that the life of the filter is extended; the continuous cavitation that occurs at the surface of the filter itself prevents blockages and/or encrustations [53].

Separation: The basis of this technology is a new principle concerning the separation of particles; the high-energy US waves applied to a low-frequency emulsion (<30 kHz) have been used to separate the emulsion into its water and oil components [54]. However, this principle must be more extensively developed before it is marketed, since high power US waves easily exert the opposite effect by inducing the formation of a more stable emulsion or dispersion. In the dairy sector, the cleaning of the membranes used for the concentration/separation of proteins is a fundamental step to maintain the permeability and selectivity of the membrane and to bring the plant back to its original capacity to minimize the risks of bacteriological contamination and produce acceptable products. As shown in a study by Luján-Facundo et al. (2016) [55], US effectively cleans membranes, and this effectiveness increases at lower frequencies.

Viscosity: Ultrasound can both increase and decrease viscosity, and depending on its intensity, the effect is either permanent or temporary [37]. In particular, US assists in processes such as homogenization, emulsification, cellular dispersion, the extraction of intracellular material, activation and/or deactivation of enzymes, maturation, drying, and degassing. Milk fermentation in the presence of bifidobacteria to produce yogurt is accelerated by US because lactose hydrolysis is enhanced by the enzyme β-galactosidase, which is released after bacterial cells are destroyed [56]. US also disperses milk fat to make yogurt more homogeneous and viscous. Milk is a colloidal system comprising fat globules suspended in an aqueous solution rich in dissolved carbohydrates, proteins and salts. Water and fat are immiscible liquids that tend to separate, and a US treatment of milk produces homogeneous and evenly distributed fat globules that allow milk to be used in various dairy products. A complete picture of our current knowledge of the application of US in food technology was presented in a review by Chemat et al. (2011) [57]. This review provides a theoretical background and some details on US, technology, techniques and food safety precautions, including processing, preservation and extraction. Therefore, some of the factors that make the combination of food and US processing one of the most promising research areas in the field of modern food engineering were also discussed. A study by Candrapala et al. (2012) [58] on the effect of US on casein and calcium showed that the casein micelles and calcium concentration did not change, whereas the milk viscosity decreased.

Antifoaming Action: The degassing and anti-foaming treatment of liquids are interesting applications of US devices. In fact, US forces the small bubbles suspended in the liquid to rise to the surface and release the entrapped gas into the environment, thus reducing the level of gas dissolved below the liquid level. Therefore, the US treatment is used to obtain carbonated drinks without foam, in fermentation systems or in other processes where the foam worsens the quality of the products [59].

Fermentation: At high intensity, US disrupts cells and/or denatures enzymes. At low intensity, it improves the mass transfer of reagents and products through the interface or through the cell wall [60]. A review by Ojha et al. [61] published in 2017 summarises key applications of high- and low-frequency US in food fermentation applications. Ultrasound has been used in these processes to either monitor the progress of fermentation or to influence its progression. Furthermore, it has also been used to eliminate microorganisms that might otherwise hinder the process [61].

Ultrasound has been used to evaluate the quality of many foods, such as meat, fish, vegetables, and dairy products, in a non-destructive manner. An increase in the US speed allows researchers to evaluate the degree of maturation of those cheeses that harden over time and simultaneously evaluate the sensory parameters and detect cracks due to anomalous fermentation [62]. The evaluation of the properties of a food mainly occurs through the analysis of the speed of sound; the US sensors allow researchers to determine the concentration of sugar in syrups and fruit juices [63] or alcohol in some beverages. On-line and continuous measurements of the alcohol content of beer during fermentation are possible with these sensors.

Enzymatic And Microbial Inactivation: Other uses have recently been identified, such as the inhibition of enzymes and the induction of oxidation reactions [64]. The promising effects of US on food preservation have recently attracted considerable interest; Knorr (2004) [38] observed a significant reduction in the abundance of *Escherichia coli* in the whole liquid egg after treatment. Generally, most microorganisms show a higher sensitivity to US at temperatures greater than 50 °C [65]. Therefore, the combination of US with a heat treatment is effective at low temperatures and subsequently improves the quality of the product [38]. US pasteurization at 50 °C has the potential to preserve the quality of many food products in terms of the physicochemical properties, colour and flavour compared with conventional pasteurisation techniques that involve much higher temperatures. The percentage of *Saccharomyces cerevisiae* cells that are inactivated by US varies depending on the amplitude of the US wave and the initial number of cells, and higher values are observed for higher amplitudes and for a low number of initial cells [66]. Furthermore, the application of US in combination with other disinfection techniques (heat, pressure, antibiotics, and UV rays) exerts a synergistic effect compared to individual treatments. Treatment effects improve with increasing intensity and decrease with increasing frequency [67].

Fresh vegetables may be contaminated when they are harvested and processed, and the contamination may persist when the vegetables are sold to a consumer. In addition, other pathogens may develop during product storage. The cutting and crushing of fresh vegetables are the main causes of microbial contamination, and the most commonly present microorganisms are *Pseudomonas* species. When plant products are harvested, they are also qualitatively modified due to respiratory activity, leading to a decrease in the carbohydrates produced by fruit enzymes and an increase in the maturation rate. The deterioration of fruits is primarily caused by the presence of amylolytic enzymes produced from the activity of microorganisms that are present on the surface of the fruit plant tissue and is affected by various factors, such as irrigation, soil, pollution, and the presence of insects and animals. Another important source of contamination is *Penicillium*, which can attack vegetables and fruits such as tomatoes, cucumbers, potatoes, and beetroots. This microorganism appears as a velvety colony with a blue-green centre and yellowish exudates and endows products with an unpleasant odour. Another contaminant is *Rhizopus*, which contaminates various vegetables and fruits, such as potatoes and strawberries, during refrigerated transport. This mould covers the surface of a plant product with pasty areas of a greyish mycelium [68].

Because washing with tap water is unsuitable for removing weeds, a thorough wash with chemical agents such as sodium hypochlorite, sodium bisulphite, sulphur dioxide, chlorine dioxide, calcium chloride, organic acids, and ozone is performed. Among these chemical agents, an aqueous solution of sodium hypochlorite is the most commonly used disinfectant. Unfortunately, sodium hypochlorite is only effective at concentrations that contain active chlorine, which damage plant quality and exert corrosive effects on equipment. Additionally, sodium hypochlorite is not applicable to organic products. This problem is more important for ready-to-eat foods, e.g., vegetables, which are consumed without cooking. In this case, microbiological decontamination of the wash water is necessary prior to its use in a working line [69].

A group of researchers assessed the suitability of high-performance US microbial disinfection and process water recycling to inactivate *E. coli* O157:H7 inoculated in water. Ultrasound was successfully applied to a water treatment process in the fruit and vegetable sector because the antimicrobial efficacy of US is not influenced by continuous variations in the process water quality. This technology is suitable for the fruit and vegetable sectors to reduce water consumption, waste water production and formation of disinfection by-products [70]. Mizrach et al. [71,72] studied the variations in the physicochemical properties of mango during the maturation and storage phases. Mango is one of the most frequently consumed tropical fruits worldwide, and the demand for mango as ready-to-eat food is increasing.

However, this fruit is subject to dulling and pulp softening. A group of researchers assessed the effect of a US treatment on the organoleptic characteristics and nutritional quality of mango and determined that the treatment preserves the consistency, colour, and content of the carotenoid phenols and ascorbic acid as well as the control product [73,74].

Sonication is also used to tenderize meat by releasing myofibrillar proteins from muscle cells and increase the water-binding capacity and cohesion of the meat. Low-intensity US is mainly applied to fresh produce, i.e., fruits and vegetables, because of its ability to penetrate a product without causing damage and its ability to reveal any defects [75]. Continuous process monitoring in gaseous, liquid or molten media is a fundamental requirement for process control. Ultrasound sensors or sensor systems contribute to the development of these quality control processes. These sensors measure the speed and attenuation of sound waves and are used to determine sugar levels in processed fruit or alcohol levels in wines and liqueurs. These sensors have also been used to monitor variations in alcoholic grade during the fermentation of wine and beer and to evaluate the quality of fruit and vegetables before and after harvesting and during storage [76,77].

## 4. Cost Reduction and Improvements in Quality with US Technology

Constant improvement in existing processes and new technologies are continuously introduced in the food sector, with the aims of improving production efficiency, guaranteeing greater safety, and quality, and reducing energy costs. Therefore, food processing technologies are a continuously developing sector. Changes in consumer tastes and the need to offer increasingly safe and higher quality products entail innovative and constantly evolving research. In fact, the introduction of new technologies may favour a reduction in processing times and an improvement in the management of processing conditions. These aspects are strictly linked to obtaining high-quality products that preserve the natural characteristics of the product as much as possible [78]. Another very important aspect that must be considered is the reduction of the energy requirements of the processes to limit both the environmental and the economic costs. Ultrasound has been applied to meat products, vegetables and fruits, cereal products, aerated foods, honey, food gels, food proteins, and food enzymes for microbial inactivation, freezing, drying, and extraction. Table 2 shows some different uses of US in terms of reducing the cost and improving the quality of food products.

In recent years, the demand for new techniques to extract chemical from solid plant matrices with shorter extraction times, reduced organic solvent consumption, and increased pollution prevention (“green” methods) has increased. Novel extraction methods include UAE [88], microwave-assisted extraction (MAE) [89], supercritical fluid extraction (SFE) [90] accelerated solvent extraction (ASE) [91] and a cyclically pressurized solid-liquid extraction with the Naviglio extractor (NE) or Rapid Solid-Liquid Dynamic Extraction (RSLDE) [92], and are fast and efficient. These techniques have the possibility of working at elevated temperatures and/or pressures, substantially decreasing the time of extraction. In particular, US processing is a novel and promising technology in the food industry [93]. Sonication is a laboratory method that uses high-frequency US delivered by a sonicator for various purposes. A sonicator is a device that generates amplified mechanical vibrations utilizing the high-frequency electrical current produced by a generator. The US waves are transmitted to a tank containing water that is set to various temperatures, and the energy effect produced by cavitation is exploited for extraction purposes. Therefore, US is used to control production processes, assess food characteristics, detect defects, control fruit ripening, improve the extraction yield and speed of food components, and evaluate the conservation state. Ultrasound has been used to improve conventional food processing unit operations by reducing energy and chemical requirements, thus offering a greener option. 

Consequently, food production processes have attracted increasing attention, and research on new technologies for improving the production efficiency, safety and quality of food while reducing energy costs is on-going. Ultrasound-mediated extraction from vegetable matrices is based on the transmission of very high-frequency vibrations at different wavelengths in an extraction batch containing the desired plant species in the extracting liquid. In particular, a frequency range of 16 kHz to 100 kHz is used, as higher frequencies would be too energetic and could degrade the active principles of vegetable matrices [22]. The extraction time ranges from a minimum of twenty minutes to a maximum of one hour and depends on the plant part that is treated, i.e., leaves, flowers, fruits, bark, and woody roots, or the consistency of the solid or liquid matrix. For solid substances, grinding is necessary to obtain particles measuring approximately 3–5 mm in size, which is the optimal size for complete extraction of the extractable substances at predetermined times. Ultrasound extraction produces water- and lipid-soluble extracts and is more advantageous than conventional methods because all active ingredients are potentially extracted simultaneously using a mixture of solvents, ensuring that the matrix is exhausted. The extraction is performed at room temperature. An increase in temperature of 1–2 °C at the end of the process does not affect the quality of the extract; in fact, the increase prevents the possible caramelization reactions of the sugary constituents that are naturally present in plant materials.

Recently, US applications in food freezing have shown promising advantages. In a study by Comandini et al. (2013) [81], the application of US during immersion freezing of potato cubes was studied, and the authors particularly focused on the effects on the supercooling process. The application of power US during immersion freezing displayed a great potential in the control of the freezing process. Therefore, the possibility of reducing the freezing time and costs, as well as the influence of US on the quality, stability and sensory properties of frozen food are critically important to the food industry and should be widely investigated [81]. US irradiation enhances the rate of heat transfer processes. Kiani et al. (2012) [83] studied the heat transfer phenomenon, mainly the heat exchange at the surface. US irradiation exerted a promising effect on enhancing the convective heat transfer rate during immersion cooling. Therefore, more investigations are required to determine the characteristics of US-assisted heat transfer and the proper method for applying US to assist in the cooling and/or freezing processes [83].

The propagation of US waves in a medium generates various physical and chemical effects, and these effects have been harnessed to improve the efficiency of various food processing operations. Ultrasound has also been used in food quality control as a diagnostic technology. An article by Cheng et al. (2015) [84] provided an overview of recent developments related to the application of US at low temperatures and closely related processes such as freezing, thawing, freeze concentration and freeze drying. The applications of high-intensity ultrasound to improve the efficiency of the freezing process, to control the size and size distribution of ice crystals and to improve the quality of frozen foods have been discussed in considerable detail. The use of low-intensity ultrasound to monitor the ice content and the progress of the freezing process has also been highlighted [84].

Freshly processed fruits and vegetables are a fertile substrate for the growth of microorganisms and often favour the development of diseases caused by the consumption of products contaminated by pathogens. The contamination of fruits and vegetables might occur at every stage, from cultivation to processing. Polluted environments during cultivation or poor hygiene conditions during processing increase the risk of contamination with pathogens. Irrigation water without the potability requirement [94] and soil contaminated with pesticides, heavy metals or urban and industrial wastewater are the main sources of pollution. Ultrasound has been used in modern continuous washing systems where fruits, vegetables and other foods typically cultivated “on the ground” as products of the agricultural sector are both washed and sorted, allowing workers to obtain a high degree of cleanliness and an excellent anti-bacterial activity that the simple system of rinsing with water does not achieve [87]. The US tanks work both at low and at high frequencies and the ceramic transducers that are currently used are very resistant to mechanical stress, ensuring the effectiveness of the cleaning action. Washing occurs with a duration of a few minutes in demineralized water, with or without the addition of small doses of detergent that is chosen based on the composition of the contaminant to be eliminated and the rapidity of the onset of cavitation, a phenomenon that creates an action for the mechanical removal of dirt at the molecular level. Every solvent used and every degree of dirt has an optimal operating temperature that considerably increases the efficiency of the wash. Ultrasound uses every surfactant molecule of the liquid, a mixture of water and detergent, allowing maximum savings in product, water and electricity consumption.

The food sector has equipment and parts composed of stainless steel and plastic materials that must comply with precise and strict rules concerning hygiene during each processing phase. Ultrasonic cleaning is particularly suitable for the final and intermediate cleaning of dirty parts, for the determination of residual dirt levels, for the cleaning of the entire machinery, for the maintenance of the plants and for the acceleration of the processes. Ultrasonic cleaning allows workers to eliminate all food residues present on the moulds of the chain for the production of cured meats, where the steel dies contain holes with very small diameters that are obstructed by the remains of the processing, in a few minutes [95].

In the poultry sector, the hygiene of the egg holder, which is frequently subject to residues of various kinds and also from dirt due to the breaking of eggs, is very important. The maximum cleaning and disinfection to eliminate remains of eggshells and other elements that might contaminate the product is possible with high power ultrasounds that act in a mixture of water and an appropriate detergent by producing a cavitational field that causes a perfect and rapid elimination of any kind of dirt from the plastic in which the individual shells of fresh eggs are packed and placed. Ultrasonic cleaning guarantees excellent protection from microbes, moulds, bad odours, and any micro-residues that are harmful to hygienic food production. Surfaces that contact food require strict sanitation procedures for decontamination from food residues and possible biofilms, and the results obtained with current technologies are not always optimal. In a study by Allen et al. (2008) [96], industrial meat and fish boxes and live chicken transport boxes naturally contaminated with food residue and effluents from a commercial crate cleaning system were immersed in an ultrasonic water bath. Enterobacteria counts were progressively reduced compared to the initial values when the immersion time increased from 0 to 120 s and the water temperature increased from 35 to 58 °C, but the action of the US on the biofilm was not very effective. However, the combination of a US treatment with an immersion temperature of 60 °C reduced the enterobacteria count to levels below the detection limit within 1 or 3 min, while the aerobic plate count was reduced after 3 min. A US treatment had a possible role in cleaning the boxes if used in combination with higher immersion temperatures and might significantly contribute to hygiene control [96]. In the dairy sector, the cleaning of the membranes used for the concentration/separation of proteins represents a fundamental step to maintain the permeability and selectivity of the membrane and to bring the plant back to its original capacity, thus minimizing the risks of bacteriological contamination and producing acceptable products. 

Ultrasonic food cutting guarantees speed and efficiency, even for foods that are difficult to process. The ultrasonic blades guarantee a clean and precise cut for cakes, bread, pizza, cheese, chocolate bars, stuffed foods such as nuts and raisins, candies, and liquorice. Moreover, the ultrasonic cutting of products composed of different layers produces a noticeable visual impact and facilitates subsequent packaging operations [79]. This technique has many benefits, including both the precision and aesthetics of the cut, as well as the speed, minimal movement of the product and less waste of the ingredients, but mainly the lack of adhesion of the various products, both hard and soft, to the blade and considerable reduction in time spent inactive to clean the blade itself. In addition, several studies are currently on-going using US to reduce meat maturation times while at the same time reducing costs [28].

In the packaging process, US technology represents the best alternative to the traditional bonding methods using adhesives and heat sealing by offering numerous advantages, including a safe, economical, hygienic and environmentally friendly processing method [97]. Ultrasound has been applied in welding to create thermoformed blister packs, polythene aluminium containers or polypropylene-coated valve-bags. The main advantage for the food industry is that the vibrations guarantee a cold welding, without the risk that the food inside the packaging sustains thermal damage [98].

Ultrasound devices are also used in molecular gastronomy, a subdiscipline of food science that studies the chemical and physical transformations that occur in food during their preparation. The objectives of molecular gastronomy are to transform the kitchen from an empirical discipline to a real science. In this case, the US waves propagate through the water to evenly reach the surface of the food subjected to the process, producing a succession of compression and decompression waves (cavitation). This process has the macroscopic effect of increasing the product temperature by approximately 5–6 degrees. The advantage should be linked to the actual lack of applied heat, which is replaced by mechanical energy, and the “massage” to which the cells of the treated product would be subjected, which would thus release more aromas and nutrients [99].

In recent years, the physical and chemical effects of US on liquid and solid media have been widely used in food processing applications. In a review by Chandrapala et al. (2015) [100], specific applications in food processing, such as emulsification, filtration, tendering and functionality modification, were highlighted and exploited the physical forces generated by US in the absence and presence of cavitation.

High-intensity US increases the efficiency and speed of extraction processes for many food components, such as oils, flavourings, pigments, and bioactive ingredients, including antioxidants and essential oils from aromatic plants such as mint, artemisia, and lavender. The crucial factors affecting high-intensity US are the high pressure and temperature applied. During cavitation, the collapse of bubbles destroys the cell walls of plant materials, releasing their contents [101]. This method has been used to obtain polysaccharide extracts [102]; bioflavonoids, such as esperidine [103]; anthocyanins [104,105]; and other substances that are used in many food supplements because of their nutritional properties.

Recent scientific studies on the applications of US for producing fermented beverages include the adaptation of US to the vinification process of grapes. In this process, an increase in the extraction efficiency of phenolic compounds from grapes is important, and US has been used to break the cell walls via cavitation [106]. The effects of high-power US on red wine polyphenols have been evaluated to improve product quality. Ferraretto et al. [107,108,109] evaluated the effects of high-power US on the phenolic structure of red wines with significant differences in the tannic fraction, and no anthocyanin degradation was observed following the treatment. By studying the effects on the raw material and during the vinification process, the researchers evaluated the effectiveness of this method for the extraction of phenolic compounds from grapes. Tests were conducted on different grape varieties, and after a few minutes of treatment at different frequencies, the extraction of polyphenolic substances increased. A reduction of up to 30% in the process duration compared with classical maceration was achieved. In addition, the effect on fermentation lees was evaluated. Ultrasound has been shown to exert an important effect on the rapid extraction of macromolecules from lees, which is useful for the colloidal and aromatic evolution of wines. Results comparable to those obtained with traditional ageing of lees with or without the addition of enzymes were obtained after a brief US treatment. Based on these results, US technology might be used to accelerate the evolution of red wines, which normally requires a long incubation in a cellar [110].

High-intensity US has antimicrobial activity and inhibits enzymatic activity [111]. The former is associated with cavitation and other phenomena that contribute to the damage of pathogen cell walls, resulting in death. US-treated microbial cells, even non-lethal species, do not regenerate, even under favourable environmental conditions. However, for food storage, the critical factors of this process are the volume of the treated material, the amplitude of the US wave, exposure time/contact with microorganisms, microorganism type, food composition, and treatment temperature. For an effective treatment, food should be exposed to high-intensity acoustic waves for long periods, which might potentially compromise the quality of the final product. An US technique is used in combination with other conventional treatments, i.e., a temperature or pressure treatment, to maintain food quality and ensure food safety [112]. This combination allows US to accelerate the food sterilization rate and reduce the treatment duration and working temperatures, resulting in a product with better organoleptic, nutritional and safety features [113]. Studies conducted by Knorr [38] in 2004 showed a reduction in the *E. coli* bacterial load in eggs after treatment with US, and the physicochemical characteristics of the product, such as colour and flavour, were superior to those observed after pasteurisation, which uses higher temperatures.

Another US application for sanitation is the treatment of barriques infested with *Brettanomyces*/*Dekkera*. A study by Yap et al. [114] compared the efficacy of US sanitization with conventional sanitation techniques. At 60 °C, high-pressure water removes between 50% and 90% of solid residues on the inner surface of a barrel, while a US treatment removes 99% of solid residues. Among the emerging technologies designed to improve sanitation and increase the shelf life of milk, US has been used alone or in combination with heat and/or pressure to inhibit microorganisms that may proliferate in products. The efficacy of US against many bacteria, including *E. coli*, *Staphylococcus aureus*, *Pseudomonas fluorescens*, *Debaryomyces hansenii*, and *Clostridium sporogenes*, and for organoleptic characteristics, such as smell and taste, has been reported. Therefore, by tuning the frequency, US has been utilized in many industrial applications, including food applications. Ultrasound techniques are relatively cheap, simple, and energy saving, and thus became an emerging technology for probing and modifying food products [115]. However, according to the literature, for an US treatment to be effective, the food should be exposed to high-intensity acoustic waves for a relatively long time, which impairs food quality. Therefore, an ideal solution might be a combination of US with conventional treatments to maintain food quality and ensure safety [116].

## 5. Conclusions

Following the first applications of US in food technology for analytical purposes, i.e., quality assessment, scholars noted that US could be used during food processing. The use of US in processing industries has steadily increased over the past several years, resulting in permanent changes in food materials in liquid systems through cavitation. Ultrasound processes activate microorganisms and enzymes to preserve or decontaminate foods, particularly when US is combined with heat and high-pressure techniques. An increasing number of industrial processes utilize US to aid in the process of mixing materials, form foams and agglomerates, precipitate dust, improve filtration efficiency, dry products, and extract solid materials and bioactive compounds from vegetables and food. Ultrasound technology can replace traditional sanitization methods, and US does not alter the organoleptic properties of foods. However, several problems remain to be explored for the further development of this technology in various sectors. More fundamental research studies are still needed to identify factors that influence the ability of power US to perform different functions. Furthermore, a better understanding of the complex physical-chemical mechanisms underlying the actions of US and its effect on the technological and functional properties of food will also contribute to strengthening the future applications of US technologies in the food industry.

## Figures and Tables

**Table 1:** Mechanical, chemical, and biochemical effects of US.

**Mechanical Effects**	**References**
Crystallization of fats, sugars, etc.	Luque de Castro et al., 2007 [19]; Cook and Hartel, 2010 [20]
Degassing and destruction of foams	Dedhia et al., 2004 [21]
Extraction of aromas	Chemat et al., 2017a [22]
Filtration and drying	Tao and Sun, 2015 [23]
Freezing	Kiani et al., 2011 [24]
Mixing and homogenization	Mason et al., 2005 [25]
Precipitation of airborne powders	Riera et al., 2006 [26]
Meat tenderization	Jayasooriya et al., 2004 [27]; Alarcon-Rojo et al., 2015 [28]
**Chemical and Biochemical Effects**	**References**
Bactericidal action	Yu et al., 2012 [29]
Wastewater treatment	Oturan et al., 2014 [30]
Modification of the growth of living cells	Guo et al., 2015 [31]
Alteration of enzymatic activity	Huang et al., 2017 [32]
Sterilization of equipment	Chemat et al., 2017b [33]; Koubaa et al., 2018 [34]

**Table 2:** Some applications of US that are of considerable interest in the food industry.

Food	Purpose	Reference
Cheese	Reduction of product losses (cut)	Schneider et al. [79]; Arnold et al., 2009 [80]
Potatoes	Reduction of structural damage (freezing)	Comandini et al., 2013 [81]
Food systems	Time saving (marinating, filtration, and oxidation)	Carrillo-López et al., 2017 [82]
Food materials	Reduction of heating and cooling costs (cooking and freezing)	Kiani et al., 2012 [83]; Cheng et al., 2015 [84]
Skim milk and sunflower oil	Shelf-life improvement (emulsification)	Leong et al., 2017 [85]
Food and agricultural products	Improvement of product structure (mixing and drying)	Musielak et al., 2016 [86]
Fruit and vegetable	Microbiological safety (anti-foaming, degassing, and sterilization)	Bilek et al., 2013 [87]
